# Agreement and Clinical Utility of the Easytone Transpalpebral Tonometer Compared with Goldmann Applanation, Tono-Pen, and Icare in Healthy Eyes

**DOI:** 10.3390/diagnostics15212766

**Published:** 2025-10-31

**Authors:** Osman Kizilay, Serap Karaca, Gokhan Celik, Omer Faruk Yilmaz

**Affiliations:** 1 Department of Ophthalmology, Zeynep Kamıl Gynecology and Pedıatrıcs Educatıon and Research Hospıtal, 34668 Istanbul, Turkey; osmankizilay@gmail.com (O.K.); gcelik279@hotmail.com (G.C.); 2 Department of Ophthalmology, Goztepe Prof. Dr. Süleyman Yalçin City Hospital, 34668 Istanbul, Turkey; dryilmazof@yahoo.com

**Keywords:** intraocular pressure, Easytone Transpalpebral Tonometer, Goldmann Applanation Tonometer, Tonopen AVIA, Icare 200

## Abstract

**Objective:** To compare intraocular pressure (IOP) measurements obtained using the Easytone Transpalpebral Tonometer (ETT) with the Icare 200 (IC200), Tonopen AVIA (TPA), and Goldmann Applanation Tonometer (GAT) in healthy individuals. **Methods**: Fifty-eight right eyes of 58 healthy volunteers underwent IOP measurement with all four devices. Three consecutive readings were taken per device and averaged. Two masked observers performed all measurements. Agreement was assessed using intraclass correlation coefficients (ICCs) and Bland–Altman analysis, and repeated-measures ANOVA compared mean IOP values. **Results:** Mean IOP values were 15.18 ± 1.88 mmHg (ETT), 14.45 ± 2.24 mmHg (TPA), 13.38 ± 2.65 mmHg (IC200), and 14.33 ± 2.03 mmHg (GAT) (*p* < 0.001). ETT provided significantly higher values than IC200, TPA, and GAT, while IC200 underestimated IOP compared with TPA and GAT. No difference was observed between TPA and GAT. Inter-observer agreement was excellent (ICC 0.805–1.000). Agreement analysis showed weaker ICC values for ETT–TPA (0.642) and ETT–IC200 (0.615). Bland–Altman plots confirmed the closest agreement between GAT and TPA, and the poorest agreement between ETT and IC200. **Conclusions:** ETT tends to overestimate and IC200 to underestimate IOP compared with GAT. TPA demonstrated the closest agreement with GAT and may be the most reliable alternative in clinical practice. ETT can be useful when applanation is not feasible, but its limited agreement should be considered.

## 1. Introduction

Intraocular pressure (IOP) is an important parameter in the diagnosis and management of glaucoma. Intraocular pressure measurement is generally performed with three different methods: transpalpebral measurement, tonometry, and manometry [1,2]. Corneal biomechanical properties are important in IOP measurement. The transpalpebral method measures IOP through the eyelid without direct corneal contact. Goldmann Applanation Tonometry (GAT), Non-Contact Tonometry (NCT), Tonopen Tonometry, Rebound Tonometry (Icare Tonometry), Perkins Tonometry, Dynamic Contour Tonometry (Pascal Tonometry), Schiotz Tonometry, and Ocular Response Analyzer (ORA) are methods that measure IOP directly through the cornea with tonometry [3,4]. Manometry (Direct Measurement) is invasive and used to obtain precise measurements of the anterior chamber during surgery [1].

All IOP measurement methods have advantages and disadvantages depending on their different working principles. GAT has been in use since the 1950s and remains the gold standard for IOP measurement today [1]. Due to the challenges associated with using GAT and the prolonged duration required for IOP measurement, alternative techniques and devices for measuring intraocular pressure have been developed. IOP measurements can be influenced by factors such as central corneal thickness (CCT) and the biomechanical properties of the cornea [5]. ETT was developed to reduce measurement errors associated with the biomechanical properties of the cornea. This portable tonometer enables IOP measurement without direct contact with the cornea [6]. Although several studies have investigated the performance of these devices separately, few have simultaneously compared ETT, IC200, TPA, and GAT in the same cohort of healthy individuals. Moreover, evidence regarding the inter-observer reliability of ETT relative to established tonometers is limited. The aim of this study was therefore to compare IC200, TPA, and GAT with ETT in healthy eyes and to evaluate their agreement and inter-observer reliability.

## 2. Methods

### 2.1. Study Population

This cross-sectional study included 58 healthy volunteers (29 women, 29 men; mean age 35.3 ± 7.9) and 58 healthy controls selected from hospital staff and patient companions between July and September 2020. All participants had normal eye examination results. Patients excluded from the study had corneal astigmatism >3 diopters, corneal pathologies (such as edema, scarring, dystrophy), a history of intraocular or refractive surgery, eyelid, conjunctival or scleral pathology, contact lens use, dry eye syndrome, glaucoma, cataract, anterior segment dysgenesis, systemic or periocular steroid use, systemic diseases that could affect corneal biomechanics, or an intraocular pressure greater than 21 mmHg. Central corneal thickness was measured using the Accutome Accupach VI pachymeter (AccuPach VI; Accutome Inc., Malvern, PA, USA), and axial length was measured using the Ellex Eye Cubed A/B Scan Biometer (Eye Cubed; Ellex Inc., Eden Prairie, MN, USA).

Intraocular pressure (IOP) was measured using four different tonometers: ETT (Easytone Transpalpebral Tonometer, JSC Yelatma Instrument-Making Enterprise, Moscow, Russia), IC200 (Icare IC200, Icare Finland Oy, Helsinki, Finland), TPA (Tono-Pen AVIA, Reichert Inc., Depew, NY, USA), GAT (Goldmann Applanation Tonometer, Haag-Streit, Bern, Switzerland). All measurements were performed in accordance with the manufacturer’s guidelines. For IC200 and TPA, disposable sterile probes or covers were used, and topical anesthesia (0.5% proparacaine) was instilled for TPA and GAT. Measurement sequence: To minimize patient discomfort and standardize the protocol, all participants underwent IOP measurement in the same order: ETT → IC200 → TPA → GAT, with a 5 min interval between devices.

The absence of randomization was acknowledged as a limitation. Number of measurements and definition of successful reading: For each device, three consecutive readings were obtained per eye, and the mean value was used for statistical analysis. A measurement was accepted if the device indicated validity according to its internal quality control (IC200, TPA) or if standard applanation/rebound criteria were met. If error signals or artifacts were observed, the measurement was repeated until three valid consecutive readings were achieved.

Observer protocol: Measurements were performed independently by two trained observers. Each observer was masked to the other’s results, and their readings were analyzed separately to evaluate inter-observer agreement.

### 2.2. Device-Specific Procedures

ETT: The device was placed vertically on the upper eyelid, 2–3 mm above the lash margin in the scleral region. The patient’s head was tilted slightly backward, kept parallel to the floor, and fixation was directed at 45°. Care was taken to avoid pressing on the globe during measurement. Because posture can influence IOP, this was considered a methodological limitation. IC200: A sterile disposable probe was positioned 4–8 mm from the central cornea while the patient fixated on a distant target. The device automatically recorded rebound velocity and provided a valid signal. TPA: After topical anesthesia, a sterile Ocu-Film tip cover was applied. The probe was gently touched to the central cornea until a valid reading was obtained. Three valid consecutive measurements were required, and the mean was used for analysis. GAT: With topical anesthesia and fluorescein dye, measurements were performed at the slit-lamp. The tonometer prism was gently advanced to applanate the central cornea, and IOP was determined when the inner edges of the semicircular fluorescein rings aligned.

In addition to the measurement protocol, the main technical and practical features of the four tonometers are summarized in Table 1. This table outlines their measurement principles, requirements for topical anesthesia or fluorescein, portability, patient positioning, repeatability, and advantages or limitations in specific clinical situations. Presenting these characteristics provides a practical framework for interpreting the comparative results obtained in this study.

### 2.3. Statistical Analysis

All statistical analyses were performed using NCSS 2007 Statistical Software (Utah, USA). Data were expressed as mean ± standard deviation (SD) for continuous variables and as frequency (%) for categorical variables. Normality of distribution was assessed using the Shapiro–Wilk test. Since all four tonometers were applied to the same eyes, comparisons of mean IOP values between devices were performed using repeated measures ANOVA.

Tukey’s Post Hoc correction was applied to control for Type I error arising from multiple comparisons. Agreement analysis between devices was evaluated using intraclass correlation coefficients (ICC), calculated with a two-way mixed-effects model, absolute agreement, average measures, and reported with 95% confidence intervals (CI). ICC values <0.70 were considered to indicate poor agreement, 0.70–0.90 moderate agreement, and >0.90 strong agreement. A Bland–Altman analysis was performed to assess the mean difference (bias) and 95% limits of agreement (LoA) between device pairs. Corresponding Bland–Altman plots were generated for each comparison (ETT vs. GAT, ETT vs. IC200, ETT vs. TPA, IC200 vs. GAT, TPA vs. GAT, IC200 vs. TPA). Regression analyses were also performed to evaluate the linear relationship between tonometers, and corresponding regression plots were generated for each device pair.

## 3. Results

A total of 58 eyes from 58 healthy participants (29 women, 29 men; mean age 35.3 ± 7.9 years) were included in the study. The mean central corneal thickness (CCT) was 542.2 ± 33.3 µm, the mean axial length was 23.5 ± 0.9 mm, and the mean spherical equivalent was –0.38 ± 0.82 D. The mean IOP values are summarized in Table 2.

### 3.1. Inter-Observer Agreement

No significant differences were observed between the two observers for IOP measurements with any of the tonometers (all *p* > 0.05, Wilcoxon test). The intraclass correlation coefficients (ICC) demonstrated strong inter-observer reliability: 0.805 for ETT, 0.910 for IC200, 0.879 for TPA, and 1.000 for GAT (all *p* < 0.001), indicating excellent consistency between observers (Table 3).

### 3.2. Comparison of Mean IOP Values

Repeated measures ANOVA showed a significant difference among the four devices (*p* < 0.001). Post hoc analysis revealed that ETT measurements were significantly higher than those of IC200 (*p* < 0.001), TPA (*p* = 0.040), and GAT (*p* = 0.001). IC200 provided significantly lower IOP values compared with both TPA and GAT (*p* < 0.001 and *p* = 0.001, respectively). No significant difference was found between TPA and GAT (*p* = 0.765) (Appendix A Table A1).

### 3.3. Agreement Analysis Between Devices

**Figure 1:**
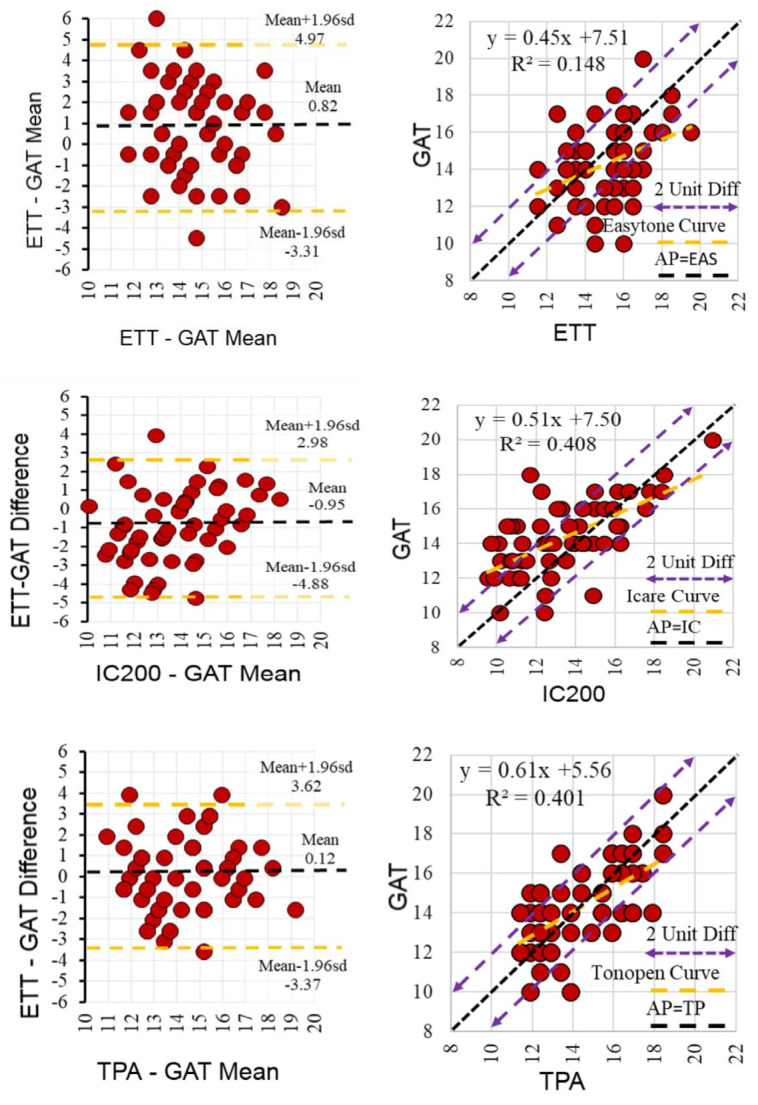
Regression and Bland–Altman analyses comparing intraocular pressure (IOP) measurements obtained with the Easytone Transpalpebral Tonometer (ETT) and other tonometers (GAT, IC200, and TPA). Left panels show regression plots (black solid line = identity line; purple dashed lines = ±2 mmHg limits), and right panels show Bland–Altman plots (mean bias = solid line; 95% limits of agreement = dashed lines).

Agreement analysis showed a moderate correlation between ETT and GAT (ICC = 0.551, 95% CI: 0.242–0.734, *p* = 0.001), while weaker correlations were found for ETT–IC200 (ICC = 0.440, 95% CI: 0.054–0.669, *p* = 0.015) and ETT–TPA (ICC = 0.455, 95% CI: 0.079–0.677, *p* = 0.012) (Table 4). Regression and Bland–Altman analyses demonstrated significant linear correlations and acceptable agreement between ETT and the other devices (Figure 1). The regression plots confirmed significant positive correlations (all *p* < 0.001). Bland–Altman plots showed that ETT slightly overestimated IOP compared with GAT and TPA, while IC200 tended to yield lower readings. The mean bias (95% limits of agreement) was −0.9 mmHg (−5.2 to 3.5) for ETT–GAT, 0.7 mmHg (−3.7 to 7.3) for ETT–TPA, and 1.8 mmHg (−2.4 to 2.3) for ETT–IC200. Overall, ETT showed stronger agreement with applanation-based tonometers than with rebound tonometry.

## 4. Discussion

In this study, ETT was compared with GAT, TPA, and IC200 in healthy individuals. GAT is still considered the gold standard for IOP measurement today. It operates based on the Imbert-Fick principle and is a non-invasive technique. Factors such as corneal biomechanics, central corneal thickness (CCT), axial length, and scleral rigidity can influence the measurement results [7,8]. It is suitable for use in patients compliant with biomicroscopic examination. However, it is not feasible for use in children, those who cannot sit upright, or under general anesthesia. Additionally, it requires topical anesthesia and fluorescein dye, making it contact-dependent. This can create a focal point for the spread of infections. The requirement for experience is also another disadvantage [9,10].

Because it may cause measurement errors in edematous or scarred corneas, alternative tonometers are gaining attention. The ETT is a portable device that eliminates cornea-related measurement errors. Its lack of requirement for topical anesthesia is among its advantages. It employs a non-invasive technique for measuring IOP through the eyelid. It is a repeatable tonometer and is well tolerated by children. However, the compression of the eyelid beneath the stick (10 g) may affect the measurement due to scleral pathologies in the area [7]. Additionally, the tone of the eyelid and the orbicularis muscle may also impact the measurement results [11]. ETT is contraindicated in cases of upper eyelid, scleral, or conjunctival pathologies, as well as in diseases or conditions that prevent the patient from maintaining a seated position. There are also other commercial models of transpalpebral tonometers available apart from ETT.

In this study, IOP measured with the ETT was statistically higher than that measured with the TPA, IC200, and GAT. We considered that this might be related to the excessive pressure applied to the eyelid. This overestimation may not be clinically significant in screening situations, but it should be considered when monitoring glaucoma patients. Similarly to the study by Montolio-Marzo et al. [12], which reported higher results with ETT compared to GAT, Del Buey Sayas et al. [13] did not find a significant difference between ETT and GAT in 73 eyes of 73 control patients. Another study, which included 26 patients with keratoconus, also reported higher IOP measurements with ETT compared to GAT [6]. We believe that the ETT can be used in screening, keeping in mind that it may yield higher IOP measurements than GAT in healthy individuals. Furthermore, there are studies indicating that other transpalpebral tonometers also produce higher measurements than GAT [14].

In this study, the Bland–Altman analysis showed that the agreement of the ETT with TPA and GAT was better than that with IC200. This suggests that ETT can be used in situations where measurement with GAT is not possible. Studies have reported that this tonometer is more comfortable for children. Iomdina et al. [2] and Montolio-Marzo et al. [12] indicated that this tonometer could be preferentially chosen for children and those with corneal irregularities. Similarly, Del Buey Sayas et al. [13] reported that the use of this tonometer provides objective measurement capabilities in patients who have undergone corneal and surface surgeries. Since this study included healthy adult individuals, we could not assess its superiority in children or patients with corneal pathologies.

In this study, the other tonometers we evaluated were the TPA and IC200. TPA is a portable tonometer that operates on similar principles as GAT but has a smaller contact area, which makes it less influenced by CCT and corneal biomechanics [15]. The IC200 rebound tonometry measures IOP based on the rebound speed of a magnetic, lightweight spherical probe against the eye’s surface [16]. It is also affected by corneal biomechanical properties. Both TPA and IC200 are portable and suitable for use in small children due to their appropriate design for use in the supine position. They are reproducible methods [17]. In this study, the average IOP measured with IC200 was statistically significantly lower than that measured with TPA. However, we found that the IOP measurements of these tonometers were consistent. Therefore, we believe that these tonometers can be used interchangeably. Lin et al. [10] have similarly noted that these tonometers can be used interchangeably in situations where GAT is not feasible.

In this study, the tonometer that showed the best agreement with the gold standard method, GAT, was TPA. No statistically significant difference was observed between the average IOP measurements of TPA and GAT. Deuter et al. [18] and Ceská et al. [19] reported that GAT and Tonopen provide similar measurements and can be used interchangeably. However, Kim et al. [20] stated that the IOP measurements with these tonometers differ and that this difference is related to age, CCT, IOP, and the diagnosis of glaucoma. The agreement between GAT and TPA in this study may be attributed to including individuals with similar CCT and IOP measurements.

Finally, while some studies comparing GAT and IC200 rebound tonometry reported that the rebound tonometer measures IOP higher than it actually is, other studies have reported lower IOP measurements [5,21]. Sachdeva et al. [22] measured IOP in 101 eyes of 101 individuals using the Icare PRO and IC200, and reported lower IOP values compared to the GAT. Similarly, in our study, we observed that the IOP measurement with IC200 was low and that its agreement with GAT was minimal. All patients included in this study had IOP measurements within the normal range, and patients with IOP above 21 mmHg were excluded from the study. Therefore, we did not compare these tonometers at higher IOP values. Additionally, body position is important during IOP measurement. Barkana et al. [23], Lee et al. [24], and Schweier et al. [25] reported that changes in IOP are related to body posture. To prevent posture-related changes in IOP, we obtained measurements of all patients in a seated position.

### Strengths and Limitations

Strengths of this study include its prospective design, the use of multiple observers to assess reproducibility, and the inclusion of Bland–Altman plots for graphical interpretation of agreement. Additionally, standardized measurement conditions were used to minimize systematic errors. Limitations include the relatively small sample size without formal power calculation, restriction to healthy individuals with normal IOP, and the absence of patients with glaucoma or ocular hypertension. Therefore, the generalizability of our results to pediatric, postoperative, or corneal pathology populations is limited. Additionally, the order of tonometer use (ETT → IC200 → TPA → GAT) was fixed and consistent. Although a 5 min interval was left between measurements, repeated tonometry may have affected subsequent readings slightly.

## 5. Conclusions

In this study, IOP measurements obtained with the Easytone transpalpebral tonometer were consistently higher than those obtained with GAT, TPA, and IC200. Among the alternative devices, TPA demonstrated the closest agreement with GAT, while IC200 tended to underestimate IOP. Although ETT showed moderate correlation with GAT, its wide limits of agreement and systematic overestimation should be taken into account. ETT may have potential as a non-contact alternative in situations where applanation tonometry is not feasible, but further studies in glaucoma, pediatric, and postoperative populations are required to validate its clinical applicability.

## Figures and Tables

**Table 1:** Main technical and practical features of the tonometers.

Feature	ETT (Easytone Transpalpebral)	IC200 (Icare 200)	TPA (Tonopen AVIA)	GAT (Goldmann Applanation)
Measurement method	Transpalpebral	Rebound	Applanation	Applanation (Gold standard)
Direct corneal contact	No	Yes	Yes	Yes
Need for an anesthetic	No	No	Yes	Yes
Need for fluorescein	No	No	No	Yes
Portable	Yes	Yes	Yes	No
Patient position	Head tilted back, parallel to the floor	Sitting or supine	Sitting or supine	Sitting at the slit lamp
Measurement time	Short (1–2 s)	Short (1–2 s)	Moderate (needs anesthesia)	Longer (requires slit-lamp setup)
Repeatability	Moderate (affected by eyelid tone)	High	High	Very high
Ease of use in children	High	High	Moderate	Low
Advantages	No corneal contact, portable, no anesthesia	Portable, no anesthesia, reproducible	Portable, less affected by CCT	Gold standard, high accuracy
Disadvantages	Affected by eyelid/scleral tone, not usable with lid pathologies	Affected by corneal biomechanics	Requires anesthesia, contact-based	Needs slit lamp, anesthesia, fluorescein, and risk of infection

**Table 2:** Demographic and clinical characteristics of participants and mean intraocular pressure (IOP) values (mean ± SD/n (%)).

Parameter	Min–Max	Median	Mean ± SD	n (%)
Age (years)	20–54	36.5	35.3 ± 7.9	
Gender				
Female Male Central corneal thickness (µm)	440–598	546.5	542.2 ± 33.3	29 (50.0) 29 (50.0)
Axial length (mm)	21.8–26.1	23.4	23.5 ± 0.9	
Spherical equivalent (D)	–2.50–+2.50	–0.50	–0.38 ± 0.82	
Mean IOP (mmHg)				
ETT IC200 TPA GAT		15.5 13.0 14.4 14.0	15.2 ± 1.7 13.4 ± 2.6 14.5 ± 2.1 14.3 ± 2.0	

SD: standard deviation; Min: minimum; Max: maximum; Median: middle value; n: number of subjects; %: percentage. ETT: Easytone Transpalpebral Tonometer; IC200: Icare 200; TPA: Tonopen AVIA; GAT: Goldmann Applanation Tonometer.

**Table 3:** Inter-observer agreement of intraocular pressure (IOP) measurements (mean ± SD, ICC, 95% CI).

Tonometer	Observer 1 (Mean ± SD, mmHg)	Observer 2 (Mean ± SD, mmHg)	*p*-Value (Wilcoxon)	ICC	95% CI
ETT	15.1 ± 2.0	15.2 ± 1.8	0.873	0.805	0.670–0.884
IC200	13.3 ± 2.7	13.4 ± 2.7	0.468	0.910	0.848–0.947
TPA	14.5 ± 2.1	14.4 ± 2.4	0.329	0.879	0.795–0.928
GAT	14.3 ± 2.0	14.3 ± 2.0	1.000	1.000	1.000–1.000

SD: standard deviation; ICC: intraclass correlation coefficient; CI: confidence interval; ETT: Easytone Transpalpebral Tonometer; IC200: Icare 200; TPA: Tonopen AVIA; GAT: Goldmann Applanation Tonometer. Wilcoxon signed-rank test and ICC with 95% CI used to assess inter-observer agreement.

**Table 4:** Agreement of IOP measurements between tonometers (ICC and Bland–Altman analysis) (Mean ± SD, ICC, 95% CI).

Comparison	ICC (95% CI)	Bias (mmHg)	95% Limits of Agreement (mmHg)
ETT vs. GAT	0.551 (0.242–0.734)	+0.9	–3.5 to +5.2
ETT vs. IC200	0.440 (0.054–0.669)	+1.8	–2.4 to +2.3
ETT vs. TPA	0.455	+0.7	–3.7 to +7.3
GAT vs. IC200	0.768 (0.607–0.862)	+1.0	–3.2 to +5.1
GAT vs. TPA	0.772 (0.620–0.867)	–0.1	–3.9 to +3.6

IOP: Intraocular pressure; ICC: intraclass correlation coefficient; CI: confidence interval; Bias: mean difference; ETT: Easytone Transpalpebral Tonometer; IC200: Icare 200; TPA: Tonopen AVIA; GAT: Goldmann Applanation Tonometer.

## Data Availability

The data supporting the findings of this study are available from the corresponding author upon reasonable request. The data are not publicly available due to privacy or ethical restrictions.

## Appendix A

**Table A1:** Post hoc multiple comparisons of mean IOP between tonometers (repeated-measures ANOVA).

Comparison	*p*-Value
ETT vs. IC200	<0.001
ETT vs. TPA	0.040
ETT vs. GAT	0.001
IC200 vs. TPA	0.001
IC200 vs. GAT	0.001
TPA vs. GAT	0.765
